# Hospital admissions and mortality due to complications of injection drug use in two hospitals in Regina, Canada: retrospective chart review

**DOI:** 10.1186/s12954-021-00492-6

**Published:** 2021-04-21

**Authors:** Polina Tsybina, Sandy Kassir, Megan Clark, Stuart Skinner

**Affiliations:** 1 College of Medicine, Regina General Hospital, University of Saskatchewan, 1440 14th Avenue, Regina, SK S4P 0W5 Canada; 2 Research Department, Saskatchewan Health Authority, Regina, Canada; 3 Department of Family Medicine, University of Saskatchewan, Regina, Canada; 4 Department of Medicine, University of Saskatchewan, Regina, Canada

**Keywords:** Injection drug use, Hospitalization, Addiction medicine, Substance use, Talcosis

## Abstract

**Background:**

Infectious complications of injection drug use (IDU) often require lengthy inpatient treatment. Our objective was to identify the number of admissions related to IDU in Regina, Canada, as well as describe patient demographics and comorbidities, yearly mortality, readmission rate, and cumulative cost of these hospitalizations between January 1 and December 31, 2018. Additionally, we sought to identify factors that increased risk of death or readmission.

**Methods:**

This study is a retrospective chart review conducted at the two hospitals in Regina. Eligible study cases were identified by querying the discharge database for predetermined International Classification of Diseases code combinations. Electronic medical records were reviewed to assess whether each admission met inclusion criteria, and hospitalization and patient data were subsequently extracted for all included admissions. Mortality data were gleaned from hospital and Ministry of Health databases. Data were analyzed using Excel and IBM SPSS Statistics to identify common comorbidities, admission diagnoses, and costs, as well as to compare patients with a single admission during the study period to those with multiple admissions. Logistic regression analysis was used to identify the relationship between individual variables and in- and out-of-hospital annual mortality.

**Results:**

One hundred and forty-nine admissions were included, with 102 unique patients identified. Common comorbidities included hepatitis C (47%), human immunodeficiency virus (HIV) (25%), and comorbid psychiatric disorders (19%). In 23% of all admissions, patients left hospital prior to treatment completion, and 27% of patients experienced multiple admissions. Female patients and those with chronic pain were more likely to be readmitted (*p* = 0.024 and *p* = 0.029, respectively). Patients admitted with infective endocarditis were more likely to die during hospitalization (*p* = 0.0001). The overall mortality was 15% in our cohort. The estimated cumulative cost of inpatient treatment of complications of IDU in Regina was $3.7 million CAD in 2018.

**Conclusion:**

Patients with history of IDU and hospital admission experience high mortality rates in Regina, a city with paucity of inpatient supports for persons who use injection drugs. Needle syringe programs, opioid agonist therapy, and safe consumption sites have been shown to improve outcomes as well as reduce healthcare costs for this patient population. We will use our findings to advocate for increased access to these harm reduction strategies in Regina, particularly for inpatients.

## Introduction

Saskatchewan, a Canadian Province with a population of 1.2 million, is leading Canada in the rate of new HIV diagnoses per capita, with 14.9 new HIV diagnoses per 100,000 persons in 2018 [1]. The province also has the highest incidence of hepatitis C virus (HCV) in Canada, with an average annual rate of 60.4 new cases per 100,000 over 2012–2016, about double the national average [2]. Injection drug use is the risk factor that unites these communicable diseases, and it is the most common risk factor for both HCV and HIV acquisition in the province [3].

There were an estimated 7300 persons who use injection drugs (PWID) in Saskatchewan in 2016, amounting to 0.99% of the population at that time [4]. Although no specific estimate is available in the literature, a large proportion of PWID living in the province reside in Regina. Regina is Saskatchewan’s capital and has a population of 261,684 [5]. As one of Saskatchewan’s two metropolitan areas (the other being Saskatoon, population 330,674), Regina likely has a greater prevalence of IDU than rural areas of the province [5, 6]. Fifty two percent of all visits to all harm reduction facilities across the province occurred in Regina Qu’Appelle region in 2017 [7]. Similarly, Regina Qu’Appelle distributed 53% of all syringes provided by harm reduction programs in the province that year.

PWID often develop infectious complications that require inpatient treatment. In the USA, several large studies have been carried out by applying international classification of diseases (ICD) criteria to data from various hospitalization databases to examine IDU-related morbidity, mortality, and costs [8–11]. These studies reveal that among IDU-related admissions, infectious complications [11], skin and soft tissue infections (SSTIs), sepsis, osteomyelitis, and infective endocarditis (IE) are most common [8, 9]. They also found high mean hospitalization costs, $55,107 USD in a Michigan study [9]. While opioids are common [11], increasing rates of stimulant [8] and amphetamine [11] use were found among PWID who were hospitalized. These studies outlined the epidemiology and high costs of IDU-related admissions in the United States.

Canadian literature on hospitalizations secondary to IDU examines smaller cohorts, with noteworthy studies conducted in British Columbia, Ontario, and New Brunswick. The Vancouver Injection Drug User Study (VIDUS) is a prospective cohort study where PWID were recruited through self-referral and street outreach since 1996. A study of 598 VIDUS participants with focus on hospitalizations was published in 2001 [12]. A total of 440 participants made 2763 visits to the emergency department (ED) over 3 years, and 210 participants required 495 hospital admissions. HIV-positive status and female sex were the only two variables associated with frequent hospital admissions. Average hospital utilization cost per patient per day was reported to be $610 CAD in 1999. Our literature review did not find other Canadian studies of costs due to IDU-related hospital admissions or ER visits. A cohort study of 663 PWID in Ottawa showed that methadone therapy and having a regular family physician were both associated with reduced ED visits [13], but we did not find other studies on factors associated with ER visits or hospitalization costs among PWID. Two case series [14, 15] examined IDU-related IE in tertiary centers in New Brunswick and Ontario, and found comorbid HCV at 69–70% in both studies, and comorbid HIV at 0–7.9%. The studies differed in which drugs PWID admitted with IE used: opioids and cocaine were most common, with amphetamines a minority in the New Brunswick study [14], but polysubstance use presided in the London, Ontario study, with sole stimulant and opioid use accounting for only 22.8% and 9.4% of cases, respectively [15]. We thus wanted to further examine, in our Canadian context, costs associated with IDU-related admissions, as well as comorbidities, protective or exacerbating patient characteristics, and prevalence of drug type used.

We also wanted to examine another locally known comorbidity that has been infrequently reported in the literature: talcosis, which is not typically included in reviews of hospitalizations of PWID [16–18]. Talcosis related to IDU is actually a misnomer, as the disease is caused not only by talc, but also by other substances found in injected street drugs as well as injected medications intended for oral use [16, 17]. Microparticles of these foreign materials become lodged in the pulmonary vasculature and cause an inflammatory reaction, granuloma formation, and, in severe cases, this leads to fibrosis. Progressive dyspnea is typically the chief symptom, and disease can progress even after cessation of IDU. Although remissions have been reported, the disease is at times fatal or requires lung transplantation [16, 18]. In our center, PWID with talcosis are seen frequently, and in our clinical practice talcosis in PWID portends a very poor prognosis.

Since the report of VIDUS hospitalizations data some 20 years ago, there have been few comprehensive studies that describe hospital admissions secondary to IDU in Canada, and none that report on related costs or include admissions due to talcosis. Our purpose is to fill this knowledge gap. We aimed to identify the cost of admissions related to IDU by finding the number of IDU-related admissions, frequency of various admission diagnoses, average length-of-stay (LOS), intensive care unit (ICU) LOS, comorbid diagnoses, reported drugs used, yearly mortality, and IDU-related readmission rate in Regina, Saskatchewan, between January 1 and December 31, 2018. Additionally, we sought to identify comorbidities, admission diagnoses and demographic characteristics that increased risk of readmission or mortality.

## Methods

This study is a retrospective chart review conducted at both Regina, Saskatchewan hospitals, the Regina General Hospital and Pasqua Hospital. The two hospitals are the only tertiary care centers in Southern Saskatchewan and together offer 639 acute care beds. This study was approved by the Research Ethics Board of the former Regina Qu’Appelle Health Region (REB-19-57). International Statistical Classification of Diseases and Related Health Problems, 10th Revision, Canada (ICD-10-CA), was used to select admissions with diagnoses of interest. We queried the discharge database for patients admitted between January 1 and December 31, 2018, whose discharge abstracts had one of the predetermined ICD-10-CA codes or code combinations (Table 1).

**Table 1:** ICD code groups used to identify admissions related to complications of injection drug use

ICD code group	ICD-10-CA codes	Description of the ICD-10-CA codes
Substance use	F11, F13, F14, F15, F16, F19	Mental and behavioral disorders due to opioids, sedatives/hypnotics, cocaine, other stimulants, hallucinogens, multiple drug use, and other psychoactive substances
Drug withdrawal or overdose	F10.3, F10.4, F11.3, F11.4, F12.3, F12.4, F13.3, F13.4, F14.3, F14.4, F15.3, F15.4, F16.3, F16.4, F17.3, F17.4, F18.3, F18.4, F19.3, F.19.4, T40, T42, T43, Y11-13	Withdrawal from various substances, overdose, or poisoning
Infectious sequelae of injection drug use	A40, A41, B24, B37.6, G06, G07, I26, I28, I33, I38, I39, I64, I65, I66, I71, I72, I74, I80, I81, I82, J85, K61, K65, K75, L02, L03, L04, L08, M00, M01, M46, M65, M86	Infectious sequela: sepsis, HIV disease, intracranial and intraspinal abscess, pulmonary embolism, other diseases of pulmonary vessels, endocarditis, stroke, occlusion or stenosis of precerebral arteries, cerebral artery occlusion, aortic aneurysm, other aneurysm, arterial embolism and thrombosis, phlebitis and thrombophlebitis, portal vein thrombosis and other venous thrombosis, embolism, lung/mediastinal abscess, anal or rectal region abscess, peritonitis, other inflammatory liver disease, cutaneous abscess, furuncle, carbuncle, cellulitis, acute lymphadenitis, other skin and subcutaneous tissue infection, pyogenic arthritis, inflammatory spondylopathies, abscess of tendon sheath, osteomyelitis
Methadone use	Z92.28	Personal history of other drug therapy (methadone)
Talc pneumoconiosis	J62.0	Pneumoconiosis due to Talc dust

Admissions were included if the discharge summary had at least one ICD code from ICD groups listed:

Substance Use AND Withdrawal or Overdose

Substance Use AND Infectious Sequela

Methadone Use AND Withdrawal or Overdose

Methadone Use AND Infectious Sequela

Talc Pneumoconiosis

**Fig. 1:**
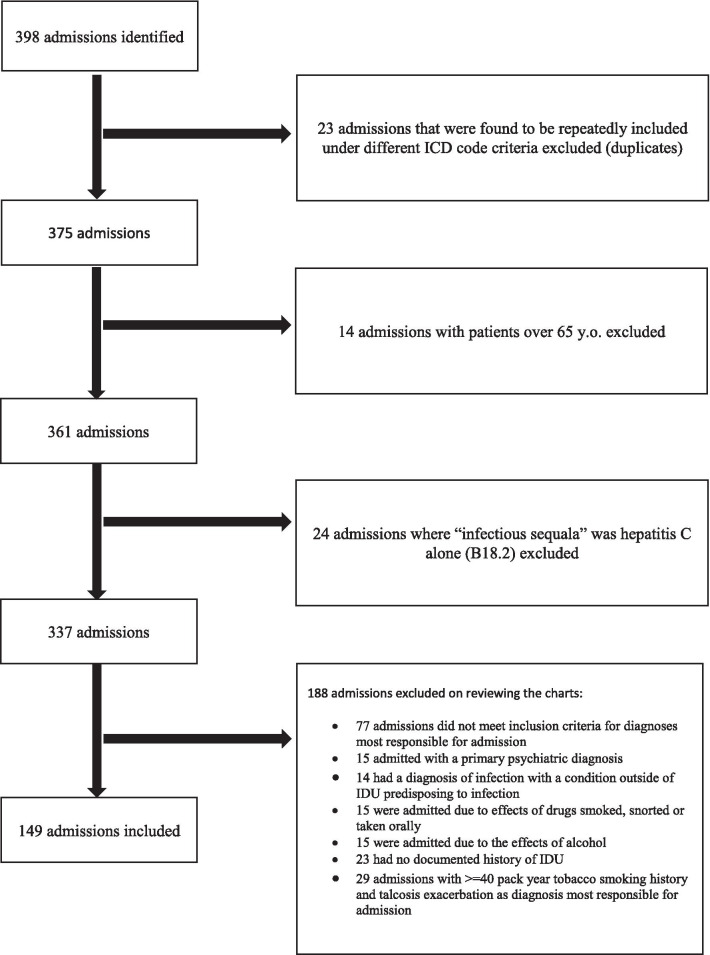
Admissions excluded

The goal of this study was to include the admissions that were specifically due to complications of IDU in Regina over the course of one year. We aimed to exclude admissions that were due to other harmful substance use, including smoking, drugs not used through injection, and alcohol. To accomplish this, we have initially started with a very broad range of ICD code combinations listed above in an attempt to capture diagnoses that may be otherwise missed with a more restrictive algorithm, such as septic emboli resulting in stroke, bacteremia resulting in aortic pseudoaneurysm, etc. Subsequently, we narrowed down the eligible cases considerably by scrupulous manual review of each electronic medical record (Fig. 1). Each admission to hospital in Regina requires a dictated admission note as well as a dictated discharge summary done by or on behalf of the most responsible physician; most consultant reports are also dictated. This documentation is available on the electronic medical record. We read these documents to obtain history of drug use, chronic comorbidities, and chief reason for admission to hospital for all patients and every admission.

Three hundred and ninety-eight admissions were identified using the ICD criteria described above. Duplicate hospitalizations were excluded. Patients under the age of 16 or over 65 were excluded to maintain specificity of the algorithm, as previously done by other groups [8]. Patients whose only infectious sequela was chronic hepatitis C (B18.2) who did not otherwise satisfy criteria as listed in the ICD groups were excluded, as it was assumed that chronic hepatitis was not causing acute symptoms and thus was not the reason for their admission. Subsequently, electronic medical record for each hospitalization remaining was reviewed. Each admission was assigned a unique study identification number, repeat admissions for the same individual were assigned an additional unique number, and de-identified patient information was then added to the database. Comorbidities, diagnoses most responsible for hospital admission, and history of IDU as well as other details of substance use history were obtained from discharge summary dictated by or on behalf of most responsible physician. If this information was not provided in the discharge summary, admission note and consultant reports from the same hospitalization were reviewed. If neither the presence nor the absence of substance use history was documented during the admission in question, all hospital documentation for this patient over the preceding 12 months was reviewed (if the patient had another hospitalization in that time). Admission notes and discharge summary notes for patients admitted with talcosis were additionally screened for tobacco smoking history. If smoking history was not listed in the notes from a hospitalization in question, all hospitalizations for the preceding 12 months were reviewed. All information obtained from the electronic chart was abstracted and entered in the database.

Hospitalizations with any of the following diagnoses listed as diagnoses most responsible for hospital admission were included: bacteremia, sepsis, IE, abscess (in any organ or system), cellulitis, carbuncle, furuncle, phlebitis or thrombophlebitis, HIV, acquired immunodeficiency syndrome (AIDS) and opportunistic infections due to AIDS, septic emboli (arterial or venous, to any organ), septic arthritis (any joint), osteomyelitis, talcosis, withdrawal, overdose, toxicity, poisoning, intoxication, any substance-induced psychiatric diagnoses. Hospitalizations where a primary psychiatric diagnosis was listed in the discharge summary as diagnosis most responsible for admission were excluded, regardless of additional diagnoses listed. This was done to avoid including admissions that can be primarily explained by a psychiatric diagnosis (ex. overdose secondary to depression) and make this study more specific to effects of IDU only. Eligible hospitalizations were then screened for diagnoses further predisposing to infection, and patients with the following were excluded: infection as a result of open fracture or other trauma, diabetic foot infection or cellulitis in a patient with diabetes, infection following a gunshot wound, and infected pressure ulcers in patients with paraplegia or quadriplegia. The team additionally planned to exclude patients with congenital immunodeficiencies as well as patients on chronic immunosuppressive agents; however, we did not encounter these among our eligible cases. Eligible cases were then screened for route of substance use, and cases where substance was identified as being used exclusively orally, through smoking, or snorting were excluded. Admissions due to effects of alcohol alone or alcohol use disorder were excluded. Finally, patients who did not have IDU documented in their electronic medical record were excluded. For patients admitted with an exacerbation of talcosis, patients with documented 40 pack year or greater tobacco smoking history were excluded, to avoid overlap with effects of chronic obstructive pulmonary disease (COPD) secondary to tobacco use.

The data analysis and statistics were carried out using Excel [19] and IBM SPSS Statistics Version 22 [20]. Average length of stay and cost of stay were obtained from the Canadian Institute of Health Information, with averages values from 2017 to 2018 used as more recent values were not available at the time of analysis [21]. Cost of each hospital stay was calculated by multiplying the resource intensity weight of the admission by average cost of standard admission in our health region. Resource intensity weights are relative weights of hospitalization cost, assigned to each patient according to the case mix group that the individual is placed in based on discharge data. The case mix group assignment is based on diagnosis most responsible for admission, comorbidities, age, complications in hospital, length of stay, and other factors, with hundreds of case groups in existence [22, 23]. Resource intensity weights are used in funding allocation, as well as estimation of cost of given hospitalizations across Canada [22, 23]. The outcomes of hospital admissions due to IDU were tabulated using descriptive statistics and then compared using Pearson’s Chi-squared or Fisher’s exact tests. Categorical variables were presented as frequencies and percentages, whereas continuous variables were presented means, medians, standard deviations, and interquartile ranges (IQR). We used logistic regression analysis to identify the relationship between individual variables, and type of admissions (single versus multiple admissions). We estimated the adjusted odds ratios (aORs) with 95% confidence interval (95 CI) while controlling for demographic variables, namely age and sex.

## Results

A total of 149 admissions are included in this chart review, with 102 unique patients admitted due to IDU complications (Table 2). Twenty-six percent of these 102 patients were admitted more than once during the year 2018. The number of men and women was roughly equal, with a median age of 37 (IQR: 32–45). About half of the patients admitted during the investigation period were in their thirties.

**Table 2:** Demographic characteristics of cohort

		Percentage of patients (%)
Number of admissions included	149	
Number of patients included	102	
Male	54	53
Female	48	47
Age groups		
17–29	16	16
30–39	49	48
40–49	25	25
50–59	11	11
60–65	1	1
Median age	37	

		Percentage of admissions (%)
Place of residence		
Regina	132	89
Outside of Regina	15	10

The most common diagnoses responsible for admission were SSTIs (30%) and bacteremia/sepsis (22%), though many patients had more than one diagnosis responsible for admission. IE (17%) and talcosis (15%) also accounted for a significant proportion of admissions (Table 3).

**Table 3:** Diagnoses responsible for admission

Diagnosis	Number of admissions	Percentage of admissions (%)
SSTIs	44	30
Bacteremia/sepsis	33	22
IE	26	17
Talcosis	23	15
Epidural abscess/diskitis	16	11
Septic arthritis	14	9
Osteomyelitis	10	7
Psychosis or other psychiatric diagnosis	3	2
Intoxication	3	2
Overdose	2	1
Withdrawal	1	1
AIDS	1	1

Common patient comorbidities included HIV (25% of patients) and HCV (47% of patients) (Table 4). Diabetes, heart failure, end-stage renal disease (ESRD), COPD, and cirrhosis, diseases commonly seen in inpatients on medical wards, were seen less frequently (1–3% for each).

**Table 4:** Comorbidities of patients

Diagnosis	Number of patients	Percentage of patients (%)
HIV	25	25
Hepatitis C	48	47
Alcohol use disorder	6	6
Comorbid psychiatric disorder outside of substance use	16	16
Talc pneumoconiosis	11	11
ESRD	2	2
Diabetes	3	3
Heart failure	1	1
COPD	3	3
Cirrhosis	2	2
Chronic pain	7	7
Other^a^	17	17

^a^16 patients who had an unspecified other comorbidity only had one. One patient had two other comorbidities

Opioids (47% of admissions) and amphetamines (35% of admissions) were the drugs that were most commonly reported to be used, though in 40% of admissions patients reported using multiple substances (Table 5). All patients included have a documented history of IDU within the year prior to admission; however, some patients used substances by more than one route.

**Table 5:** Drugs used

Substance	Number of admissions	Percentage of admissions (%)
Opioids	70	47
Methamphetamines	52	35
Methylphenidate	22	15
Cocaine	30	20
Benzodiazepines	8	5
Unreported	20	13
Multiple drugs reported	60	40

Of 39 patients who reported using methamphetamines, 15 (39%), 8 (20%), 3 (8%), and 2 (5%) also reported using opiates, cocaine, benzodiazepines, and methylphenidate. Additionally, of 50 patients reporting opiates use, 9 (18%), 3 (6%), and 1(2%) also reported using cocaine, methylphenidate, and benzodiazepines, respectively.

The median length of stay was 7.7 days (IQR: 4.75–13.70), with 31 admissions requiring transfer to the ICU (21% of all admissions) for a total of 188 ICU days (8% of total days). There were 22 surgical interventions. Average cost of stay was $24,982 CAD, amounting to a total cost of $3,722,332 CAD. The median cost of stay was $12,351 CAD (IQR 4174–28,446). Cost of a standard hospital stay was $7,359 CAD in the Regina Qu’Appelle Health Region in 2017–2018, by comparison [12]. In 23% of all admissions, the patient left prior to treatment completion (patient-directed discharge, PDD).

The 102 patients of this cohort required 22 surgical or interventional radiology interventions during the investigation period: incision and debridement [6], joint arthroscopy [4], spine surgery [3], Interventional Radiology abscess drainage [3], valve replacement [2], amputation [1], Interventional Radiology embolization [1], and tracheostomy [1].

Of 102 included patients, 26.5% (27) patients had multiple IDU-related admissions of whom the majority resided in Regina (92.6%). Specifically, 17 of these patients had 2 admissions each, 4 had 3 admissions and 4 had 4 admissions throughout 2018. There was one individual with 5 admissions and another individual with 7 admissions throughout the year. The median age of the patients with multiple admissions was 38 years old (IQR: 33–42). The median length of stay in hospital was 8.7 days (IQR: 5.3–11.6). A total of 36% of patients with multiple admissions (n = 27) experienced PDD. Among these 27 patients, there were 8 surgical interventions. In terms of comorbidities, among patients who were admitted more than once approximately 15% (4) had chronic pain, 15% (4) had psychiatric disorders, 22% (6) had talcosis, and 22% (6) and 48% (13) had HIV and HCV, respectively.

Patients with a single admission during the year were compared with patients who had multiple admissions. Women were 3.0 times more likely to be readmitted compared to men (95% CI: 1.2–7.5) (*p* = 0.024). Patients with chronic pain were 4.2 times more likely to experience multiple admissions compared to others (95% CI: 3.9–8.6) (*p* = 0.029). No other comorbidity predicted multiple admissions throughout the year. Yearly mortality was higher for patients who were admitted more than once compared to patients with a single admission (22% versus 12%), though this was not statistically significant (*p* = 0.215).

Eleven patients died in hospital (11%) and another four patients died in 2018 in the community, totaling a cumulative mortality of 15%. The median age of the deceased patients was 37 (IQR: 32–39) years, which is comparable to the median age of this study cohort. Out of the patients who died, 10 were women, but female sex was not found to be a risk factor for mortality (*p* = 0.160) (aOR = 2.6, 95% CI: 0.81–4.2). Patients who died in hospital were significantly more likely to be admitted with IE compared to patients who survived, and endocarditis was implicated in 8 of 11 deaths (*p* = 0.0001) (aOR = 4.2, 95% CI: 2.1–5.8). One patient with IE underwent medically assisted death. Two patients died due to advanced lung disease associated with talcosis. One patient died due to sepsis and associated multiorgan failure.

Multivariable logistic regression analysis was used to analyze the relationship between patient status of being alive or deceased and readmissions, drug use, comorbidities, admission diagnoses, and discharge conditions (PDD or regular). Death was more likely if IE was one of the admission diagnoses (*p* = 0.019), increasing risk of death by a factor of 2.4 (95% CI: 2.1–7.2). Skin and soft tissue infection was also found to be associated with risk of death (*p* = 0.036), increasing risk by a factor of 12.42 (95% CI: 11.82–13.37). No specific comorbidity increased the likelihood of death. When specific substances used were run as independent covariates, there was no statistically significant relationship between the substance used and mortality. Similarly, whether patients were admitted once or multiple times did not amount to any statistically significant difference to risk of death. Lastly, leaving hospital against medical advice did not result in increased likelihood of death (Table 6).

**Table 6:** Associations between mortality and drugs used, gender, comorbidities, and diagnoses (controlling for age and sex)

Characteristic	Patient status aOR (95% CI) Deceased REF (*n* = 15)
Single vs multiple admissions	
Multiple	1.85 (0.54–6.32)
Single	REF
Drugs used	
Cocaine	3.36 (3.17–5.50)
Methamphetamines	4.01 (3.73–9.09)
Methylphenidate	1.17 (0.37–3.42)
Opioids	1.20 (0.32–4.53)
Comorbidities	
Talc pneumoconiosis	1.71 (0.53–6.02)
Diabetes	4.61 (2.79–6.16)
Comorbid Psychiatric Disorder outside of substance use	1.76 (0.63–5.74)
HCV	1.32 (0.77–6.32)
HIV	0.58 (0.11–3.04)
Diagnoses Responsible for Admission	
SSTI	**12.42 (11.82–13.37)**
Bacteremia/sepsis	0.55 (0.48–2.84)
Talcosis	1.5 (0.43–2.67)
Psychosis or other psychiatric diagnosis	1.4 (0.66–1.58)
Endocarditis	**2.4 (2.11–7.23)**
Discharge	
PDD	1.55 (0.97–1.81)
Regular discharge	REF

Bolded values refer to *p* values < .05

aOR represent analyses adjusted for all variables presented

REF represents the variable responses used as reference in the regression analysis

## Discussion

This study shows that hospitalized PWID in Regina experience significant morbidity and mortality. Patients who experienced PDD, patients with chronic pain and women were more likely to be readmitted, so more strategies to prevent PDD and specific interventions for women and people with chronic pain who use injection drugs may improve outcomes. The considerable costs of inpatient care for this population at $3.7 million CAD, which this study likely greatly underestimates, could be reduced with a number of proven outpatient harm reduction interventions that are underutilized in our region.

The 15% mortality seen in our cohort is considerably higher than that reported in other studies examining hospitalizations related to IDU [9, 24, 25]. For example, a study from Florida reported that 4.9% of patients admitted with sequela of IDU died during hospitalization over a one-year period [25], although their study included emergency department presentations without hospital admission, so likely had a healthier cohort, and they only included in-hospital mortality, while we examined mortality both in- and out-of-hospital. Inclusion of patients with talcosis likely further increases our reported mortality in comparison with other studies, as these patients are not typically included. Moreover, PWID are a very heterogenous population and specific drugs, method of drug use, and harm reduction techniques can affect complications. For example, a significant increase in endocarditis amongst those who use extended-release hydromorphone has been reported, as it enhances survival of *Staphylococcus aureus* in drug preparation equipment [26, 27]. As such, more detailed analysis of specific drug use and technique may provide additional reasons for higher mortality in our study.

We are aware of only one review that details costs of hospitalizations for patients with complications of IDU on an institutional level, at Jackson Memorial Hospital in Florida [25]. Our literature review did not reveal a comparative study done in Canada; hence, we are using the Florida review for comparison, recognizing that there are significant differences between the USA and Canada when it comes to healthcare delivery and costs. The Florida review reports costs amounting to $11.4 million USD over one year, but their facility has more inpatient beds and a more populous catchment area than Regina does. Their median cost of hospitalization was $39,896 USD, which is equivalent to some $52,500 CAD. Our median hospitalization cost is much lower at $12,351 CAD. This may be due to private healthcare and thus higher cost of inpatient and outpatient care in the USA in general [28]. Our study differed significantly in mortality and IDU-related admission costs from Florida review.

Vancouver’s VIDUS hospitalization study identified that female sex was a predictor for frequent hospital admissions, and women who use injection drugs in our cohort were likewise at increased risk of repeat admissions [12]. This reflects that women represent an especially vulnerable group among PWID and face additional barriers to care [29, 30]. Most of the patients who died in our cohort were also women (10 out of 15 deaths). This finding was not statistically significant, but this could be due to our small sample size. Our data suggest female PWID are at high risk of death if admitted and more supports for women who inject drugs should be developed in our health region and across Canada.

In 23% of all admissions, discharge was patient-directed (PDD) and 26% of patients in our cohort had multiple admissions in 2018. Studies have shown that patients who leave prior to treatment completion have higher rates of readmission and higher mortality [31–33]. Stigma, discrimination by hospital staff, and hospital restrictions are commonly cited reasons for PDD [34, 35]. We also speculate that in our hospitals, the lack of access to supervised consumption sites (SCSs), needle syringe programs (NSPs), and inpatient opioid agonist therapy (OAT) contribute to patients leaving before treatment completion. A study conducted in Vancouver with a hospitalized cohort of 181 people with HIV who use illicit drugs showed that PDD is significantly less likely if patients utilize a specialized HIV-care center located within immediate vicinity of St. Paul’s Hospital [36]. Reducing stigma associated with IDU in Regina hospitals, as well as providing access to NSPs, OAT and SCSs can likely reduce the number of PWID who leave Regina hospitals prior to treatment completion.

NSPs, OAT, and SCSs can also reduce costs of inpatient treatment of PWID. Although Regina has three sites offering NSPs, their hours are quite limited, with only one NSP open on Saturdays and none on Sundays. There is no NSP on site or within immediate vicinity of either hospital. Numerous studies have shown NSPs to be cost-effective and efficacious in reducing transmission of HIV specifically [37–42]. Our study highlighted Saskatchewan’s increased HIV prevalence: we found a higher comorbidity rate of HIV at 25%, compared to 0–7.9% in other Canadian studies [14, 15]. As IDU is the most common form of transmission of HIV in Saskatchewan, these interventions are crucial in reducing HIV infections, SSTIs, and associated costs in our province. Uptake of harm reduction initiatives, including NSPs, is associated with reduced SSTIs in the PWID population [43]. This data suggests that NSP would not only help reduce HIV transmission, but may also affect the rates of hospitalization for SSTIs related to IDU in Saskatchewan. SSTIs was the most common reason for admission in our cohort at 30% of admissions, in keeping with other studies [8, 9]. SSTIs were also the admission diagnosis most strongly associated with risk of death, at an odds ratio of 12.42. The cost of running a NSP is estimated to be $700 to $2000 USD per client per year, which compares very favorably with the downstream costs of IDU hospitalizations we illustrate in this study [44].

As there is no Addiction Medicine service at either hospital in Regina, only patients already receiving OAT as outpatients typically have access to OAT in hospital (The licensing laws allow OAT prescribed outside the hospital to continue in hospital.) [45]. This is unfortunate, as hospital admission can be a great opportunity to start a patient on opioid substitution, as well as connect them with community resources. OAT has been proven to reduce illicit opioid use [46], decrease the incidence of HIV by up to 54% [47, 48], and decrease health service utilization [48]. SCSs have similarly been shown to be cost-effective [49, 50], decrease mortality [50], and reduce IDU [51, 52]. Canada currently has 38 operating SCSs [53]. Saskatchewan’s first SCS opened its doors in Saskatoon earlier this year [54].

This study illustrates that there are many persons in Regina who require inpatient care due to downstream effects of unsafe IDU, and SCSs could potentially prevent many of these hospitalizations, as well as reduce all-cause mortality for this patient population. While we conservatively estimate that some $3.7 million CAD was spent on hospital admissions related to IDU in the city of Regina alone in 2018, only $562,000 was spent on harm reduction strategies across the whole province of SK over the 2017–2018 fiscal year [7]. Considering these costs, our government should invest more in preventative interventions for PWID.

This study has several limitations. ED visits are not included in this review, but represent a significant cost and burden to the healthcare system [12]. ICD codes for drug use are only included in discharge abstracts by coding analysts if responsible physicians document that drug use is relevant to a given hospital admission (Personal communication from Health Information Management Systems analysts and management, Regina, Saskatchewan Health Authority), which means a number of hospital admissions that were related to IDU were missed by our review. Our estimate of the number of admissions relating to IDU is conservative because of the stringent inclusion criteria, particularly documentation of IDU within a year prior to admission in dictated notes available on the patient’s electronic medical record. However, many patients do not disclose history of IDU due to associated stigma, and some physicians do not always document history of substance use, let alone its route. While we have been able to report on the kinds of substances patients use with some detail, we do not, unfortunately, have the same detail on the route of use. Although all patients included have a documented history of IDU, some of the substances we list were likely used orally, smoked, and potentially through multiple routes. Finally, our review includes only 102 patients and thus is likely underpowered to detect differences between some of the patient groups.

## Conclusions

Hospital admissions for patients with history of IDU are associated with high yearly mortality and significant healthcare costs at a calculated $3.7 million CAD in 2018 in Regina, Saskatchewan. Making harm reduction initiatives available to inpatients would likely improve patient outcomes as well as decrease associated healthcare costs. In particular, we advocate for availability of NSPs, OAT, and SCSs to both inpatients and outpatients in Regina. An Addiction Medicine consult service would be an optimal vehicle to provide NSP and OAT to inpatients. A dedicated multidisciplinary team would not only treat opioid use disorder and teach safe injection, but also educate other healthcare staff working with this vulnerable population, reduce stigma, and hopefully reduce patient-directed discharges.

## Data Availability

The datasets used and analyzed during the current study are available from the corresponding author on reasonable request.

## Abbreviations

AIDS: Acquired immunodeficiency syndrome
COPD: Chronic obstructive lung disease
ED: Emergency department
ESRD: End-stage renal disease
HCV: Hepatitis C virus
HIV: Human immunodeficiency virus
ICD: International Statistical Classification of Diseases and Related Health Problems
ICU: Intensive care unit
IDU: Injection drug use
IE: Infective endocarditis
IQR: Interquantile ranges
LOS: Length of stay
NSP: Needle syringe program
OAT: Opioid agonist therapy
PDD: Patient-directed discharge
PWID: Persons who use injection drugs
SCS: Supervised consumption site
SSTI: Skin and soft tissue infection
VIDUS: Vancouver Injection Drug User Study
